# Lifelong Expression of Apolipoprotein D in the Human Brainstem: Correlation with Reduced Age-Related Neurodegeneration

**DOI:** 10.1371/journal.pone.0077852

**Published:** 2013-10-22

**Authors:** Ana Navarro, Elena Méndez, Celso Diaz, Eva del Valle, Eva Martínez-Pinilla, Cristina Ordóñez, Jorge Tolivia

**Affiliations:** Instituto de Neurociencias del Principado de Asturias (INEUROPA), Dpto. Morfología y Biología Celular, Facultad de Biología y Medicina, Universidad de Oviedo, Asturias, Spain; Rutgers University, United States of America

## Abstract

The lipocalin apolipoprotein D (Apo D) is upregulated in peripheral nerves following injury and in regions of the central nervous system, such as the cerebral cortex, hippocampus, and cerebellum, during aging and progression of certain neurological diseases. In contrast, few studies have examined Apo D expression in the brainstem, a region necessary for survival and generally less prone to age-related degeneration. We measured Apo D expression in whole human brainstem lysates by slot-blot and at higher spatial resolution by quantitative immunohistochemistry in eleven brainstem nuclei (the 4 nuclei of the vestibular nuclear complex, inferior olive, hypoglossal nucleus, oculomotor nucleus, facial motor nucleus, nucleus of the solitary tract, dorsal motor nucleus of the vagus nerve, and Roller`s nucleus). In contrast to cortex, hippocampus, and cerebellum, apolipoprotein D was highly expressed in brainstem tissue from subjects (N = 26, 32−96 years of age) with no history of neurological disease, and expression showed little variation with age. Expression was significantly stronger in somatomotor nuclei (hypoglossal, oculomotor, facial) than visceromotor or sensory nuclei. Both neurons and glia expressed Apo D, particularly neurons with larger somata and glia in the periphery of these brainstem centers. Immunostaining was strongest in the neuronal perinuclear region and absent in the nucleus. We propose that strong brainstem expression of Apo D throughout adult life contributes to resistance against neurodegenerative disease and age-related degeneration, possibly by preventing oxidative stress and ensuing lipid peroxidation.

## Introduction

Apolipoprotein D is a glycoprotein first isolated from the plasma HDL fraction and also found in smaller amounts in VLDL and LDL. The amino acid sequence shows a high homology with the lipocalin family but not with other apolipoproteins. The main role of Apo D is still uncertain, but by analogy with other lipocalins it may serve as a transporter of small hydrophobic molecules between the circulation and tissue [1]. The gene for human Apo D (at 3p14.2) was cloned and sequenced in 1986, and mRNA expression subsequently demonstrated in many tissues [2]. 

The Apo D protein has since been detected in many organs, tissues, and body fluids of both humans and other species [3], and is probably able to interact with a variety of different ligands depending location and physiological requirements. Adrenal gland and kidney express the highest levels of Apo D mRNA, and expression is also intense in pancreas, placenta, spleen, lung, ovary, testis, brain, and peripheral nerves. Lachrymal secretions, axillary apocrine gland secretions, urine, cerebrospinal fluid (CSF), and perilymph and middle ear fluid also contain Apo D [3]. At the cellular level, Apo D is commonly expressed by fibroblasts, especially those close to vessels [4]. 

In the human peripheral nervous system (PNS), Apo D is synthesized and secreted by fibroblasts and Schwann cells under basal conditions and upregulated during regenerative processes that follow injury. Following sciatic nerve injury, Apo D mRNA and protein expression levels increase to levels much higher than all other apolipoproteins [5,6]. Like Apo E, Apo D is synthesized locally, while Apo AI and Apo A-IV are derived from the bloodstream [5]. Peripheral nerve injury is accompanied by the release of hydrophilic molecules initially deposited in Schwann cells, macrophages, and neurilemmal cells. Cholesterol, together with other lipids, is then used for restructuring and membrane biogenesis. Apolipoproteins contribute to the mobilization of lipids and cholesterol homeostasis and have complementary roles in nerve injury in the absence of other apolipoproteins [7].

The human central nervous system (CNS) expresses a unique Apo D isoform with lower molecular weight (29 kD) than that founded in CSF or plasma (32 kD). In humans and other mammals, immunohistochemical and hybridohistochemical techniques have demonstrated Apo D on the surfaces of glial cells (astrocytes, oligodendrocytes, and microglia), pial cells, perivascular cells, and neurons [4,8–13]. 

Apolipoprotein D expression is more intense in white matter than grey matter due to particularly strong expression in oligodendrocytes, where it may be involved in myelination [7]. On the contrary, expression is lower in neurons and astrocytes and more variable across brain regions [8,9]. Furthermore, the synthesis of Apo D by astrocytes is elevated by activation in response to injury, termed reactive astrogliosis [10]. The fact that Apo D localizes to ependimocytes, glial cells, neuropil, and perivascular cells suggests that it could be a bidirectional carrier of small molecules through the blood-brain barrier (BBB) and (or) blood-CSF barrier (BCB) [8,9,14]. 

Increased expression of Apo D in the CNS has been observed in neurodegenerative and neuropsychiatric disorders (for review see 15,16). In some of these diseases, such as Alzheimer's disease (AD) and multiple sclerosis (MS), elevated Apo D expression has also been observed in the cerebrospinal fluid and plasma [17,18]. Furthermore, the correlation between regional Apo D expression pattern and sensitivity to neural pathology associated with these disorders suggests that Apo D is a marker for brain structures prone to neuropathology, such as the frontal cortex, entorhinal cortex, and hippocampus in AD, pre-frontal cortex in schizophrenia, and temporal cortex in bipolar disorder [17,19,20]. There has been little work on Apo D expression in mid- or hindbrain structures such as the pons and medulla, regions with distinct anatomical structures and rudimentary functions essential for life.

 In this study, we examined the expression of Apo D in brainstem extracts by slot-blot and in fixed sections by immunohistochemistry. Tissues were obtain from subjects of a broad range of ages to examine age-related expression changes in both whole brainstem and 11 well defined nuclei of the human medulla oblongata and pons.

## Materials and Methods

### 1. Human tissues

Use of human brain tissues were approved by “Comité Ético de investigación Clínica Regional del Principado de Asturias” as follows. These studies were granted waivers of consent on the following bases: 1) samples were gathered retrospectively from pathology archives of necropsies performed for diagnostic purposes; 2) patient identities were anonymized and completely delinked from unique identifiers; and 3) there was no risk to the participants.

All human brain tissue samples were provided by the Department of Pathologic Anatomy of the Central Hospital of Asturias and obtained from necropsies within 6 h of death. These samples were the same as those used in previous studies by our group [8-10,21]. Twenty-six brainstems from men 32−92 years old were examined. None of the patients presented a previous inner ear or neurological disease. In all cases, macroscopic and microscopic examination revealed the absence of acute, chronic, localized, or diffuse brain pathology. Tissue blocks were cut into 1 cm slices perpendicular to the brainstem axis. Two samples were obtained from each case; one was frozen at -80 ° C for protein extraction and the other fixed in paraformaldehyde (in 0.1 M phosphate buffer, pH 7.4) for neuropathological diagnosis and immunohistochemical studies. Due to the structure of the nuclei studied, samples for slot blots were not separated into white and grey matter sections. The present study was conducted according to the Declaration of Helsinki and was approved by the “Comité Ético de investigación Clínica Regional del Principado de Asturias” (Spain).

### 2. Protein slot-blotting

Apo D levels were quantified using a slot blot technique [9]. Samples from human breast cyst fluid were also included as a positive control for Apo D detection, and ExtrAvidin-Alkaline Phosphatase (Sigma, E26366) was used as an internal control. Briefly, 0.06, 0.12, 0.25, 0.5, 1.0, and 2.0 μg samples of total protein were slot blotted under vacuum in a Bio Dot apparatus (Biorad). Nitrocellulose membranes were then immunostained for Apo D protein according to the following protocol. Non-specific binding was blocked by incubation of membranes with 1% bovine serum albumin (BSA). Blocked membranes were then incubated for 1 hour at room temperature with a primary antibody against human Apo D (1:10,000; a gift from Dr. Carlos López Otín, Departamento de Bioquímica y Biología Molecular, Universidad de Oviedo) [8,22-24]. After 3 washes of 15 min each in PBS, membranes were incubated for 30 min at room temperature in biotinylated monoclonal anti-rabbit IgG (1:10,000; Sigma, B 5283), washed again as before, and then incubated with ExtrAvidin Alkaline Phosphatase (1:10,000; Sigma, E26366). After three washes of 15 min each in PBS, enzyme activity was measured by incubation in Sigma Fast BCIP/NBT solution (Sigma, B5655) for 2 hours at room temperature.

The blots of Apo D were dried and bands quantified as relative optical densities (arbitrary units) using a digital scanner (Nikon AX-110, Nikon) and ImageJ 1.37c (National Institutes of Health, Bethesda, MD, USA) [9].

### 3. Cytoarchitectonic staining and Immunohistochemistry

After fixation, tissue blocks were washed in distilled water, dehydrated, cleared in butyl acetate, and embedded in paraffin. Sections were cut at 10 μm thickness and mounted on "SuperFrost Plus" (Mentzel-Glasse) slides, dried at 36 °C for 24 h, deparaffined in xylene, and partially rehydrated by incubation in a graded alcohol series (2 min per concentration). In each case, every 80th or 160th section was stained by the Nissl/Myelin technique [25] and examined for myelocytoarchitecture. The brainstem nuclei examined were the principal inferior olivary nucleus (IO), the hypoglossal nucleus (XII), the oculomotor nucleus (III), the facial nucleus (VII), the Roller nucleus (RN), the dorsal motor nucleus of the vagus nerve (DX), nucleus of the solitary tract (NST), inferior vestibular nucleus (IVN), medial vestibular nucleus (MVN), lateral vestibular nucleus (LVN), and superior vestibular nucleus (SVN). The boundaries of these nuclei were defined according to [26] as in previous studies by our group [27,28].

For immunohistochemistry, fixed sections were permeablized with triton X (0.1%, 5 min), washed in distilled water, treated with H_2_O_2_ (3%, 5 min) to quench endogenous peroxidase activity, washed in distilled water, and treated with PBS. After blocking non-specific binding by incubation in BSA (30 min), sections were incubated with the antibody against human Apo D (1:2,000) overnight at 4 °C. Immunoreactivity was detected using the Extravidin biotin peroxidase staining kit (Sigma Extra 3), and peroxidase activity visualized by incubation with Sigma Fast DAB (Sigma D4168) at room temperature for 30 min. Finally, the sections were counterstained using a modified formaldehyde thionin method [29], dehydrated, cleared in eucalyptol, and mounted with Eukitt. For controls, representative sections were processed in the same way with a non-immune serum or specifically absorbed sera in place of the primary antibody. Under these conditions, no specific immunostaining was observed.

### 4. Quantification of Apo D Expression

For quantification of Apo D expression, the chromogenic signal was selected using Adobe Photoshop CS 8.0.1 (Adobe Systems Inc., CA, USA) and density measured by ImageJ 1.37c (National Institutes of Health, Bethesda, MD, USA) according to a procedure developed by our group [30]. Briefly, sections were photographed with a digital camera and the images imported into Adobe Photoshop. For subsequent comparison of Apo D expression in different sections, the magnification of images must also be equal. The analysis protocol is as follows: (1) To select the specific chromogenic signal, choose “Color range” in the “Select menu” of Photoshop, and with the “Eyedropper” tool click on any object in the image displaying the desired color/chromogen and all areas within the selected color range will be highlighted in an automatically generated clone image. The tolerance bar of the color range tool makes direct visualization of the selected regions possible with minimal variations in the chromogen selection. Once chromogen selection is finished, the profile used can be archived for use with similar stained sections (2). Close the “Color range” panel and all immunopositive objects in the original image appear highlighted (3). Open the “Edit” menu of Photoshop, select “Copy”, open a “New file”, and “Paste” the selected image. The new image shows only the positive selected areas on a white background (4). Convert the image to greyscale so all selected marked areas are represented in grey tones correlated with chromogen intensity. This greyscale picture must be saved in ‘tif’ format, which can then be opened in Scion Image (or other free image analysis programs such as ImageJ) (5). Open the picture in this program and calculate the “Mean density” under “Uncalibrated” conditions. The measurements obtained show the signal strength and can be copied and exported to a spreadsheet (i.e., Excel). The signal strength varies between 0 and 255 (value 0 corresponds to a nonstained section (white), and 255 corresponds to a fully stained section (black).

### 5. Statistical analyses

All statistical calculations were conducted using SPSS 15.0 for Windows. Data sets were first tested for normality using the Kolmogorov-Smirnov test with Lilliefors correction. Expression levels of Apo D were compared among nuclei by the Mann-Whitney U test due to non-homogeneity of variance. A P value < 0.05 was considered statistically significant. 

## Results

### 1. Apo D protein levels

**Figure 1:**
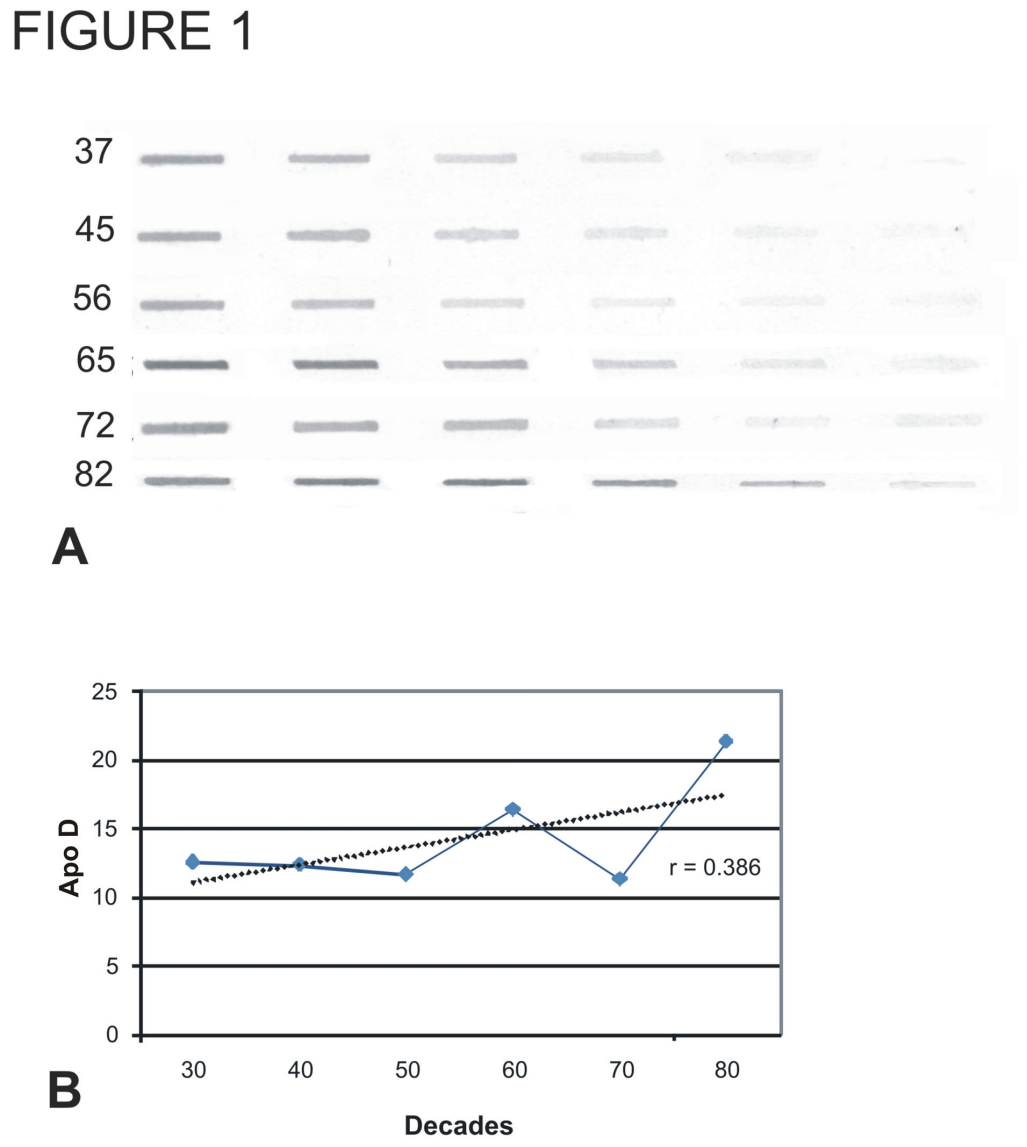
Slot blot analysis and amount of Apo D protein along different ages and decades. A) Samples from medulla oblongata of different subjects during aging. B) Amount of Apo D in samples from subjects between 35 and 82 years old. The graphic shows the densitometric analysis of slot blots. Data presented as means of relative optical density (ROD). Each point in the graph represents mean density ±standar error of the mean. Regresion lines and Pearson`s correlation coefficient (r) is also shown.

The levels of Apo D expression were measured in 26 human brainstem samples from patients ranging in age from 32 to 96 years. Densitometric analysis of slot blots showed robust and relatively stable expression throughout adult life, particularly between the forth and eighth decades (Figure 1). Regression analysis showed no statistically significant increase in expression with age.

### 2. Tissue distribution of Apo D protein

**Figure 2:**
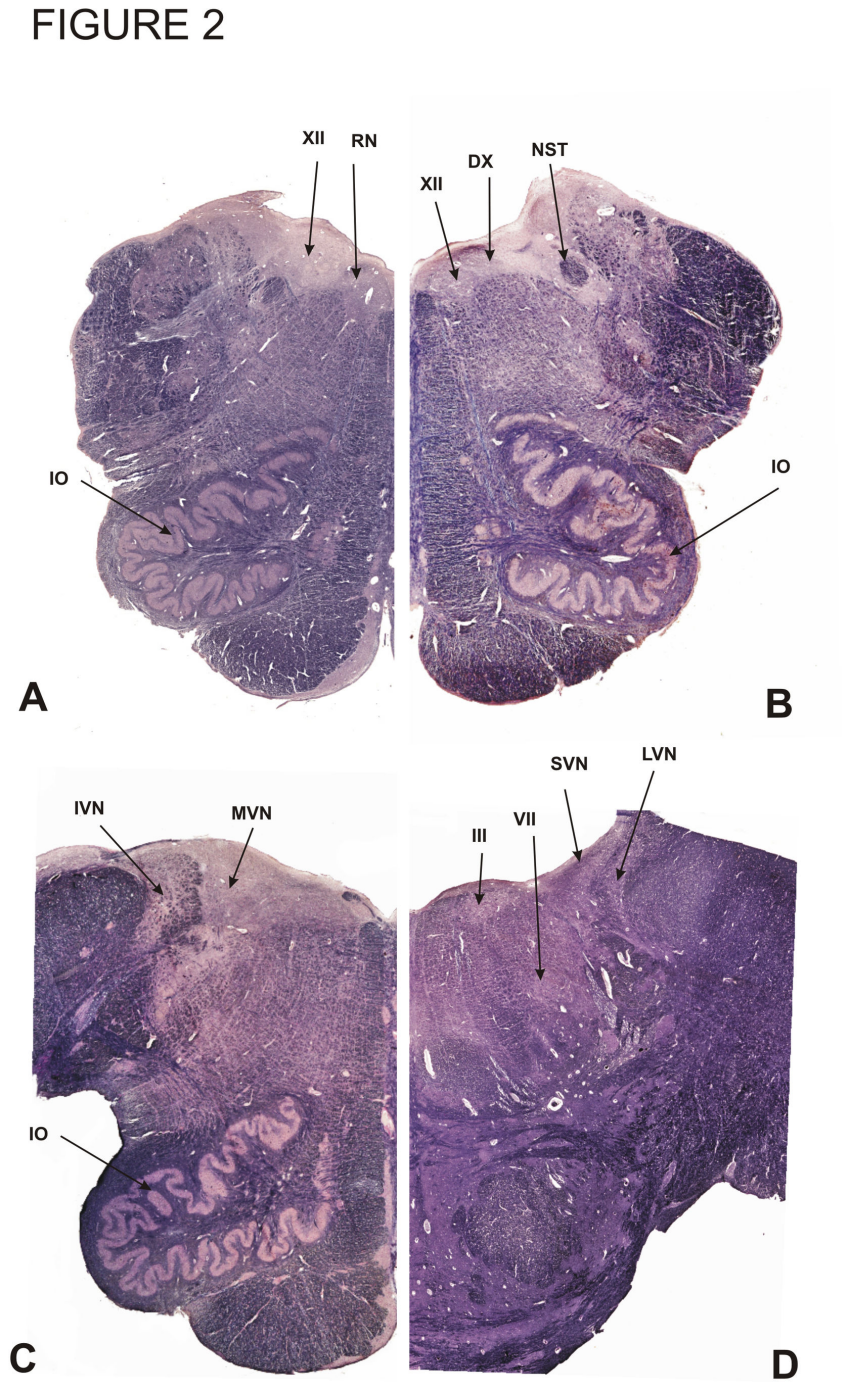
Hemisections of medulla oblongata. Four levels of medulla oblongata (A,B,C,D), stained with a Nissl/Myelin technique, with the localization of the different nuclei studied in the present paper are showed.

Expression of Apo D was also measured at the cellular level in 11 pontine and medullar nuclei of both sensorial and motor functional systems (Figure 2). All special somatosensory nuclei studied are components of the vestibular nuclear complex (VNC). In addition, we assessed Apo D expression in the inferior olivary nucleus (IO), which does not belong to either the general or special somatosensory system but has somatosensory functions (Figure 2). We also studied the hypoglossal nucleus (XII), oculomotor nucleus (III) and the facial motor nucleus (VII) as examples of somatomotor and branchimotor nuclei. The nucleus of the solitary tract (NST) and the dorsal motor nucleus of the vagus nerve (DX) were studied as examples of visceromotor nuclei. Finally, we studied a small motor nucleus located in the middle reticular substance named Roller`s nucleus (RN).

#### 2.1. Somatomotor and branchimotor system nuclei

**Figure 3:**
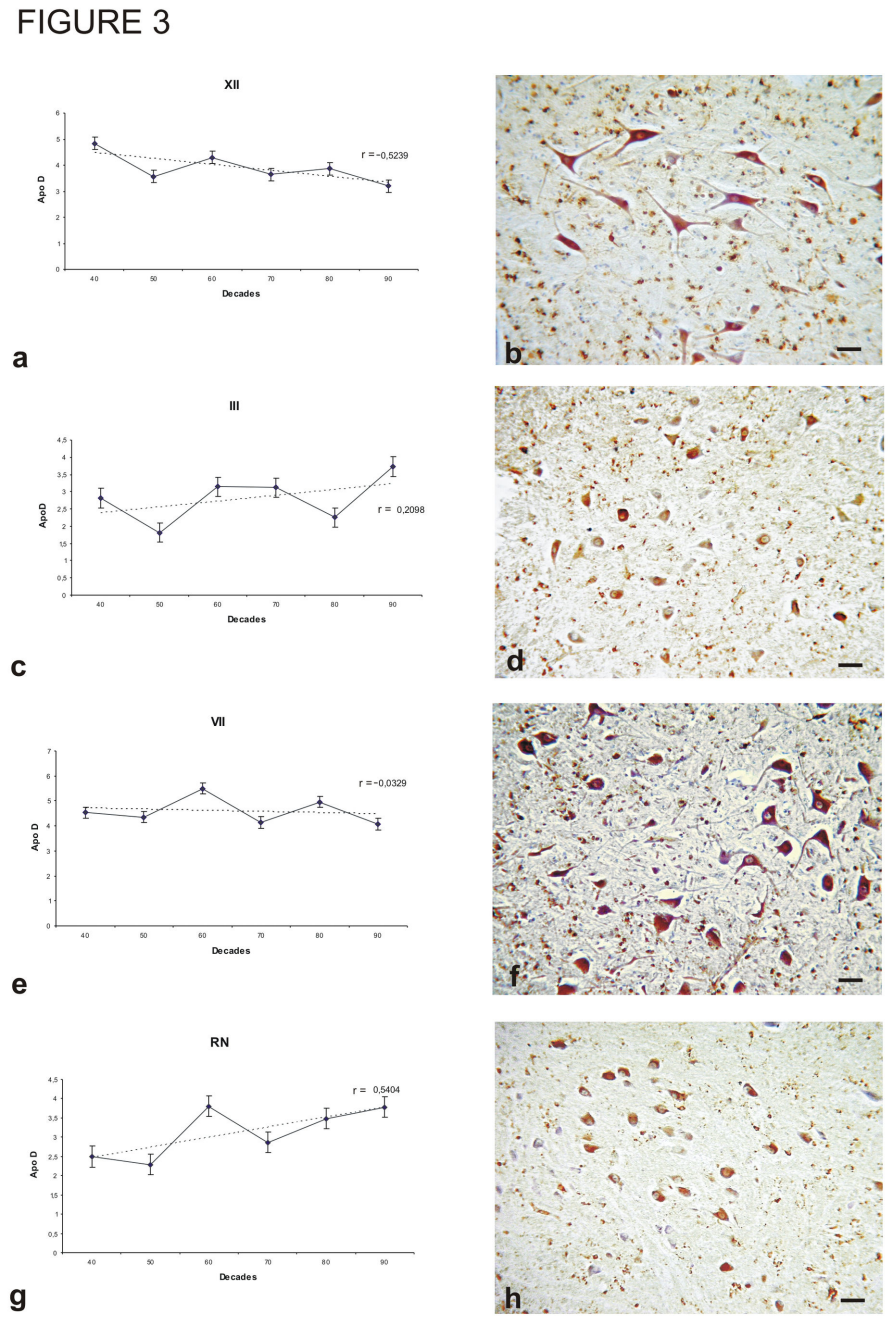
Immunohistochemistry for Apo D and amount of Apo D protein in some nuclei along different decades. A, B) Hypoglossal nucleus (XII). C,D) Oculomotor nucleus (III). E, F) Facial motor nucleus (VII). Roller`s nucleus (RN). Data presented as means of relative optical density (ROD). Each point in the graph represents mean density in a x20 field ±standar error of the mean. Regresion lines and Pearson`s correlation coefficient (r) are also shown. Bar: 40 μm.

All somatomotor and branchimotor system nuclei studied showed robust Apo D immunoreactivity throughout life. The hypoglossal nucleus (XII), located in the middle and lower third of the medulla oblongata, is part of the somatic motor column of the brainstem. This somatomotor nucleus (Figure 3A, B) exhibited the highest level of Apo D staining after the facial nucleus (Figure 3E, F). In XII, protein expression was not evenly distributed but rather located principally in larger neurons (Figure 3B). There was no significant relationship between Apo D expression and age, but there was a modest tendency for total protein expression to diminish in older subjects (r = -0.5239; p > 0.05).

The oculomotor or *abducens* nucleus (III) is also part of the somatic motor column of the brainstem and shares many characteristics with XII (Figure 3C, D). The III also exhibited intense Apo D immunoreactivity, but expression was heterogeneous, with the middle third showing the greatest protein expression, coinciding with the highest cell density. There was no significant relationship between Apo D expression and age, but there was a slight tendency for total protein expression to increase (r = 0.2098; p > 0.05). As in the other nuclei studied, Apo D was present in almost all neurons of medium and large size.

The facial nucleus (VII), a nucleus with well described topographic organization, contained many large Apo D-positive neurons and showed the highest Apo D expression of all nuclei studied (Figure 3E, F). The density of stained cells was highest in the middle third of the nucleus, decreasing in the rostral and caudal ends. There was no change in Apo D expression with age (r = -0.0329; p > 0.05; Figure 3E). 

The immunoreactivity of Roller’s nucleus (Figure 3G, H) was also very high despite being smaller than the other nuclei studied (Figure 3G, H). We found no significant relationship between age and total Apo D expression in RN, but there was a slight tendency to increase with age (r = 0.5404; p > 0.05).

#### 2.2. Viscerosensory and visceromotor system nuclei

**Figure 4:**
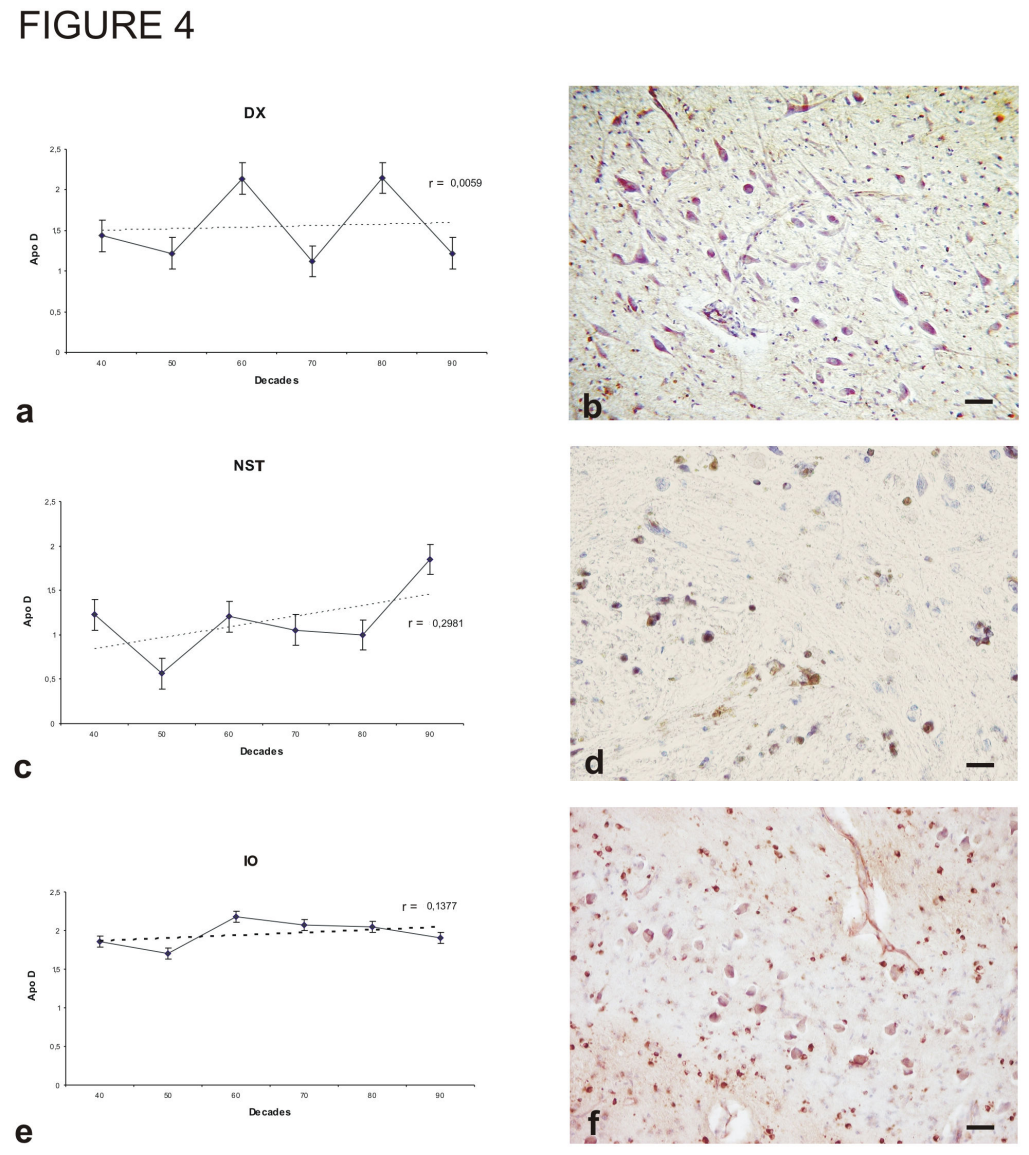
Immunohistochemistry for Apo D and amount of Apo D protein in some nuclei along different decades. A, B) Dorsal motor nucleus of the vagus (DX). C,D) Nucleus of solitary tractis (NST). E, F) Inferior olivary nucleus (IO). Data presented as means of relative optical density (ROD). Each point in the graph represents mean density in a x20 field ±standar error of the mean. Regresion lines and Pearson`s correlation coefficient (r) are also shown. Bar: 40 μm.

**Figure 5:**
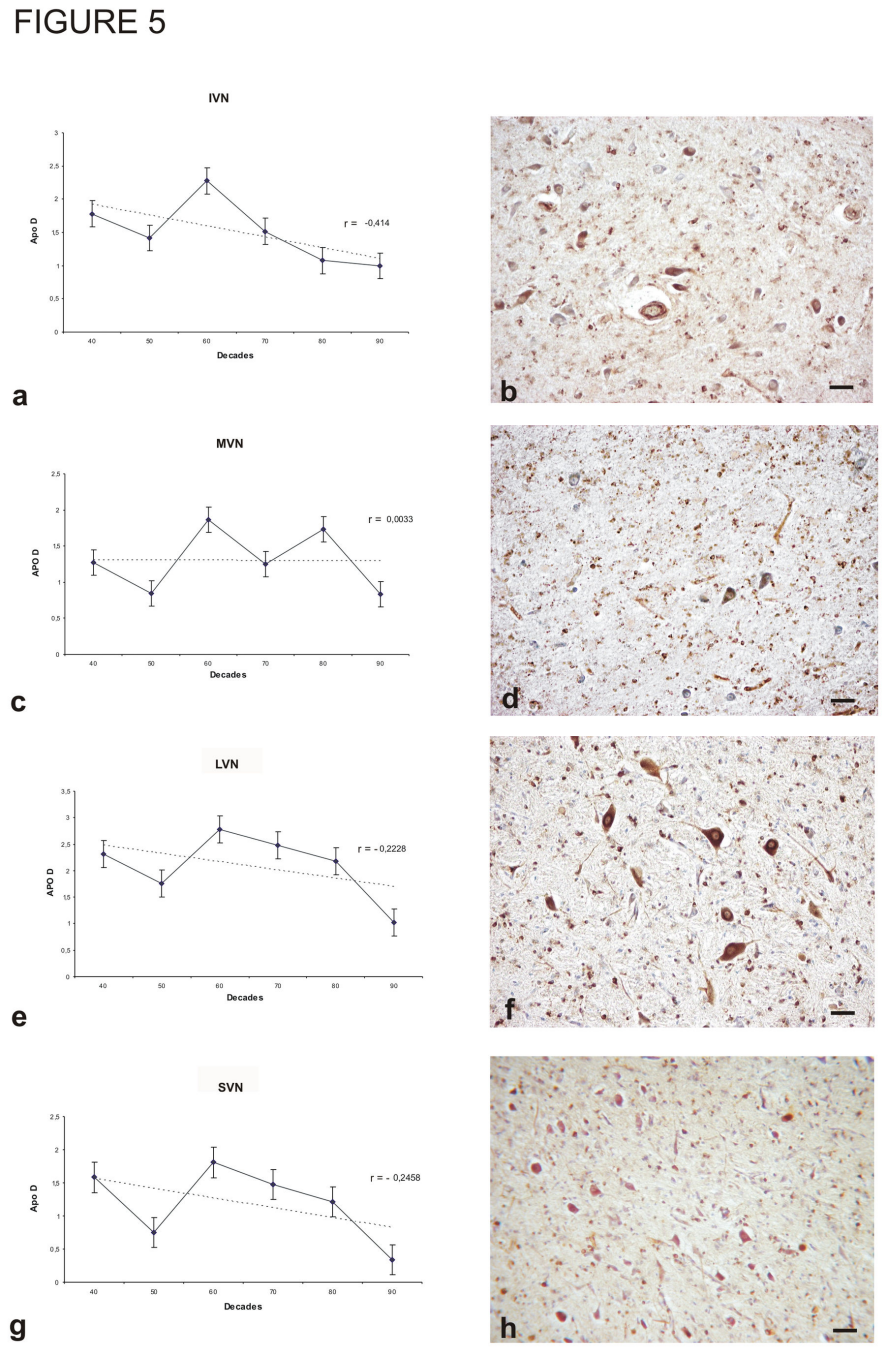
Immunohistochemistry for Apo D and amount of Apo D protein in vestibular nuclear complex along different decades. A, B) Inferior vestibular nucleus (IVN). C,D) Medial vestibular nucleus (MVN). E, F) Lateral vestibular nucleus (LVN). G, H) Superior vestibular nucleus (SVN). Data presented as means of relative optical density (ROD). Each point in the graph represents mean density in a x20 field ±standar error of the mean. Regresion lines and Pearson`s correlation coefficient (r) are also shown. Bar: 40 μm.

**Figure 6:**
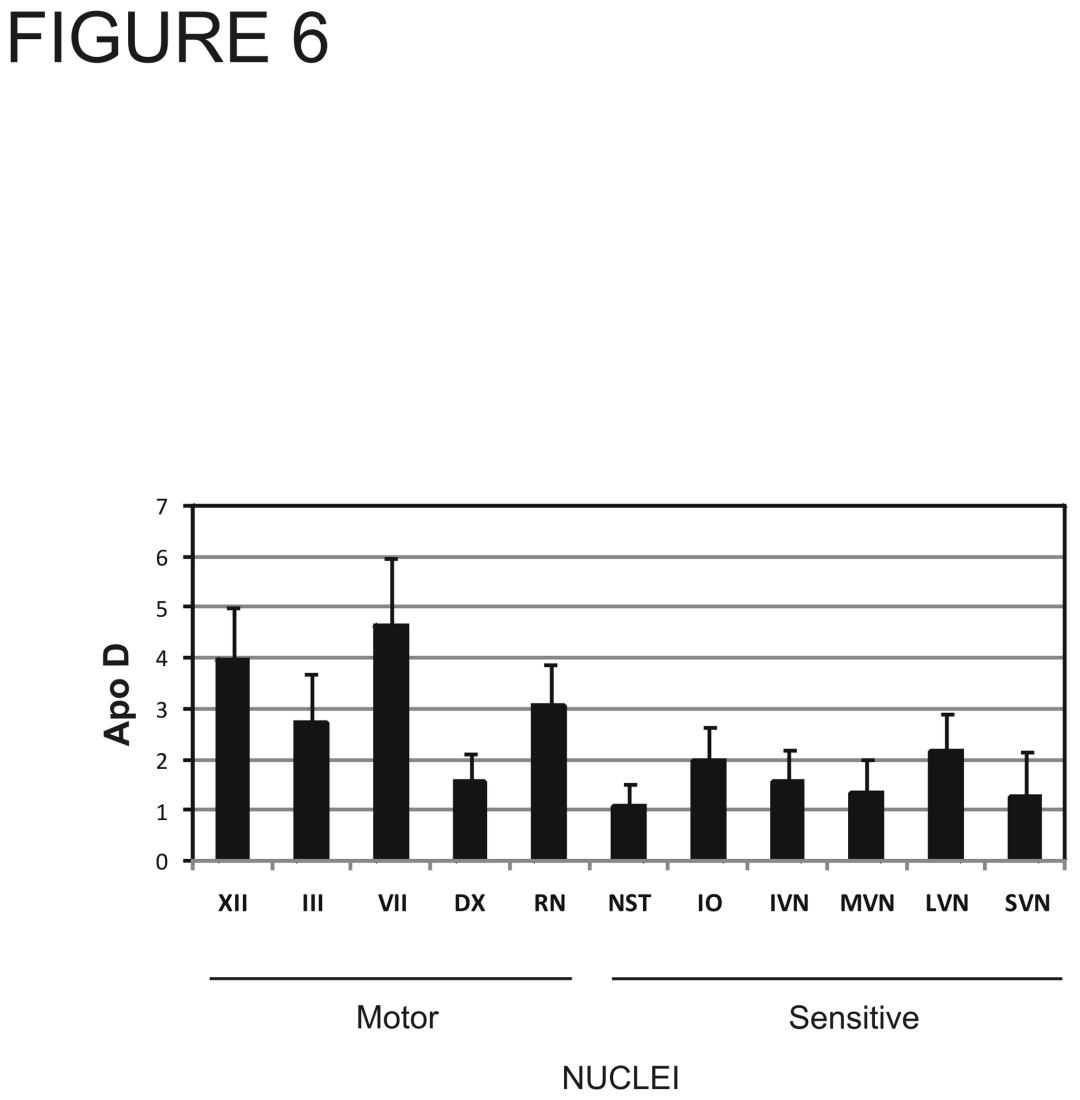
Mean of Apo D protein during aging in each nuclei of medulla oblongata studied. Nuclei are grouped according their sensory or motor function. Motor nuclei show higher quantity of Apo D than sensory nuclei. Data presented as means of relative optical density (ROD). Bars represent mean value of Apo D along all decades.

The DX (Figure 4A, B) and NST (Figure 4C, D) were studied as examples of visceromotor nuclei. Both exhibited low intensity immunostaining compared to the somatomotor and branchiomotor nuclei, and showed no statistically significant changes with age (r = 0.0059; r = 0.2981; p > 0.05). In general, these low levels of Apo D expression mirrored other sensory nuclei (Figure 5), with all showing lower immunoreactivity than somatomotor nuclei (Figure 6).

#### 2.3. The inferior olive and somatosensory system nuclei

The general and special somatosensory nuclei of the brainstem are located in the pons and medulla oblongata. We also studied IO (Figure 4E, F) due to its somatosensory functions and because the cytoarchitectural features can be easily delimited. The mean densitometric value of Apo D expression in the IO was intermediate between the motor nuclei (with generally high expression) and the sensory nuclei (with generally low expression). There was no statistically significant relationship between the densitometric value of Apo D staining and age, but there was a small tendency for increased expression with age (r = 0. 1377; p > 0.05).

The VNC consists of inferior (IVN), lateral (LVN), medial (MVN), and superior (SVN) components. The IVN contains neurons of a broad range of sizes, but medium-size neurons are predominant (Figure 5A, B). The IVN and LVN had the highest levels of Apo D expression in the VNC. In the IVN, Apo D expression was located only in the cytoplasm of larger neurons. No statistically significant relationship between age and Apo D expression was found in the IVN, although there was a modest trend for a decrease with age (r = -0.414; p > 0.05). The MVN is the largest nuclei of the VNC and densely packed with intermediate-size neurons. Despite the high cell density, Apo D expression was relatively low. Staining was preferentially located in neurons of greatest size (Figure 5C, D). Densitometric analysis revealed no significant changes in expression with age (r = 0.0033; p > 0.05). The LVN exhibited the highest Apo D expression in the VNC but again with no significant age-related changes (r = -0.2228; p > 0.05). In the LVN, all giant neurons (Deiters’ cells) showed high levels of Apo D expression regardless of subject age (Figure 5E, F). The SVN consists of large neurons arranged in nests among myelinated fibres that cross the nucleus as well as smaller isolated neurons (Figure 5G, H). The SVN expressed the least amount of Apo D of all VNC components and also showed no statistically significant changed in expression with age (r = -0.2458; p > 0.05).

Unlike forebrain regions and cerebellum, we found intense Apo D expression in many neurons and glial cells in young human brainstem nuclei. Immunostaining for Apo D was usually restricted to the neuronal cytoplasm, with highest intensity in the perinuclear region. When the signal was strong, it tended to fill the entire cell, even the neurites, but has been not observed in the cell nucleus. In general, larger neurons showed more intense staining for Apo D than smaller neurons. The number of Apo D-positive glial cells appeared highest at the periphery compared to the core of these nuclei (Figures 3-5). Finally, motor nuclei exhibited higher Apo D expression than sensory nuclei (p < 0.006). Again, expression level was independent of age (Figure 6). The physiological implications of these observations required further study.

## Discussion

In the present study, we found that expression of Apo D in medulla oblongata and pons is already high in young subjects, contrary to other areas of the brain previously studied [8,9], and widely distributed among the different neuronal nuclei in this region. We examined expression at the cellular level in eleven such nuclei and found robust expression in neurons, glial cells, and neuropil even in young brainstem. This is also in contrast to human cerebral cortex, where little Apo D is localized to neurons and appears in only a few astrocytes [8,9]. The region-specific distribution of Apo D expression was first studied in detail by our group [8] and later reported by others [19,20]. We previously demonstrated differences in Apo D expression among neuronal nuclei located in the same brain region [24] or among neurons of the same area [23]. Moreover, the absence of Apo D expression appears to enhance neuronal vulnerability to death [23,24]. Thus, we consider these results significant for future pathological studies on brainstem in various neurological diseases. The pattern of Apo D expression in human brain coincides with other lipocalins that are lipopolysaccharide (LPS)-inducible [31,32]. The highest level of lipocalin2 (LCN2) was found in the olfactory bulb, followed by the brainstem, cerebellum, hypothalamus, and thalamus, with lowest levels in the striatum, hippocampus, frontal cortex, and somatosensory cortex of normal mouse brain [33].

In human frontal cortex, expression is higher in white matter than grey matter due to higher synthesis by oligodendrocytes [8,9,13,34]. Whether this is also true in brainstem remains to be determined as the complex distribution of nuclei and fibre bundles does not allow for easy separation of white and grey matter for lysate preparation. Nonetheless, we noted the most intense expression in neurons, particularly larger neurons of somatomotor nuclei.

Increased expression of Apo D in the CNS has been associated with aging, neurodegenerative diseases, and neuropsychiatric disorders. However, the majority of studies on Apo D have focused on cerebral and cerebellar cortices and the hippocampus, areas particularly prone to pathological changes with age and during the progression of neurodegenerative diseases. In all these areas, Apo D expression in young subjects was lower in grey matter than white matter, and increased in both tissues with age and following injury. Apo D expression has also been linked to cellular senescence and growth arrest in cell culture [13,35], and expression appears correlated with age in tissues as diverse as hair follicles and the CNS [14]. Even a previous study by our group found that Apo D protein and mRNA increased during normal development and aging in frontal cortex [9]. Moreover, a similar age-dependent expression was demonstrated in the frontal cortex of rats and monkeys [36,37]. By contrast, slot blots and densitometric quantification of Apo D expression revealed that Apo D expression in brainstem ​​is already high in young to middle-age adults with no history of neurodegenerative disease or brain injury. Moreover, expression was relatively stable during aging, a result inconsistent with current assumptions that Apo D is an “age-related” protein. 

The underlying molecular mechanisms of CNS aging include instability of nuclear and mitochondrial genomes, neuroendocrine dysfunction, oxidative stress, altered calcium metabolism, and inflammation-mediated neuronal damage [38]. A growing body of evidence points toward oxidative stress caused by excess reactive oxygen species (ROS) production as one of the primary determinants of age-related neural damage [39]. Stressors that cause an extended growth arrest, such as UV light or hydrogen peroxide, increase Apo D expression [32]. In fact, the promoter region of the human Apo D gene contains several stress-activated regulatory elements like serum, acute phase, and stress response elements (SRE, APRE, or STRE) [40]. Several studies postulated that Apo D could have an important function in cellular defence against age-associated oxidative stress (OS). Indeed, studies on the fly homologue and the Apo D null mouse [41,42] showed reduced tolerance to the ROS generator paraquat (PQ) in mutant/null genotypes, an effect attributed to the control of lipid peroxidation. Conversely, transgenic animals overexpressing human Apo D [43,44], cells transfected with the gene for Apo D [20], or the direct addition of exogenous Apo D [32,45] protected against oxidants. A recent paper on PQ-treated cerebellum from transgenic mice supports the hypothesis that this lipocalin is necessary for a proper CNS response to physiological and pathological OS. The absence of Apo D modified the response of genes related to OS management or myelination, while overexpression of human Apo D in neurons almost completely abolished the early transcriptional response to OS [42]. 

We observed strong Apo D expression in all studied nuclei of the medulla oblongata and pons from both young and old subjects, and no statistically significant increase in Apo D expression with age. Thus, Apo D is not a marker of aging in the brainstem. Due to these high levels of Apo D, we speculate that human brainstem is not exposed to damaging OS during aging, that Apo D expression in this brain region is uniquely regulated, or that the predominant role for Apo D in this brain region is distinct from that in other regions. It has long been known that Apo D is critical for remyelination of rat sciatic nerve, as evidenced by the > 40-fold increase in expression in regenerating nerve compared to non-injured control nerve [6]. Elevated expression of Apo D has been found in endoneural fibroblasts of regenerating crushed nerve in rats [6]. Since Apo D is released into the extracellular environment, Spreyer et al. [6] proposed that Apo D participates in intraneural lipid transport and (or) re-utilization for the biosynthesis of membranes. In mouse peripheral nerve, Apo D is expressed by Schwann cells, by satellite cells, and by cells located between the Schwann cells [34]. We demonstrated that Apo D facilitates the recovery of locomotor function after injury, promotes myelin clearance, and regulates the extent of angiogenesis and the number of macrophages invading the injury site in mouse crushed sciatic nerve. Moreover, the regeneration and remyelination of axons were delayed in the absence of Apo D and stimulated by excess Apo D [7]. In the present study, we found that motor nuclei have higher levels of Apo D expression than sensory nuclei. We argue that this may be because motor nuclei require a greater capacity for myelination and maintenance of axonal transport.

Finally, Apo D may also regulate neuritogenesis and synaptogenesis. Co-cultures of adipocytes with peripheral neurons showed enhanced neurite and synapse formation. It has reported that apolipoproteins D and E3 exert neurotrophic and synaptogenic effects in dorsal root ganglion cell cultures [46]. Considering these results together, we suggest that Apo D is critical for neural function and homeostasis. The brainstem exhibits stable expression of Apo D throughout life due to its critical importance for survival. This brain region contains centers that regulate several functions vital to survival, including heartbeat, breathing, blood pressure, digestion, swallowing, and vomiting. It is also responsible for stimulating the reticular formation, a region involved in regulation of sleep/waking patterns and reflexes for maintaining muscle tone, posture, and gaze. 

The effects of aging on the CNS have been studied for more than a century, but only a few works have focused on effects to the brainstem. We previously reported that aging is not associated with neuronal loss in brainstem nuclei [28], only a shift from larger to smaller neurons and an increase in glial cell number [27]. Indeed, most studies of the brainstem have found little neuronal loss and no significant functional impairment. We previously described a statistically significant neuron decrease in the VNC during aging, but only in the MVN [28], which could account for the small deficits in balance observed in older individuals. One interesting result of the present study was that the MNV, the only nucleus of the VNC that loses neurons during aging, had the lowest Apo D expression of all VNC nuclei. That basal Apo D expression is higher in brainstem than in grey matter areas that lose neurons during aging and are generally more sensitive to oxidative stress [8,21,23,24] strongly suggests that the preservation of brainstem nuclei during aging is directly related to stable expression of Apo D.

## Conclusions

Although the full spectrum of Apo D functions is unclear, the current study strongly suggests that this molecule is critical for resistance to age-related neural damage. Neurons that do not synthesize or import Apo D from surrounding glial cells are more vulnerable to age- and disease-related death. Diseases or injuries of the brainstem region are normally incompatible with life, requiring stable basal expression of Apo D (and possibly other neuroprotective factors). The results of this study support the hypothesis that Apo D is a critical neuroprotective molecule against neurodegenerative disease and the ravages of age.
